# Add-On Therapy with Traditional Chinese Medicine Improves Outcomes and Reduces Adverse Events in Hepatocellular Carcinoma: A Meta-Analysis of Randomized Controlled Trials

**DOI:** 10.1155/2017/3428253

**Published:** 2017-06-07

**Authors:** Zongguo Yang, Xian Liao, Yunfei Lu, Qingnian Xu, Bozong Tang, Xiaorong Chen, Yongchun Yu

**Affiliations:** ^1^Shanghai Municipal Hospital of Traditional Chinese Medicine, Shanghai University of Traditional Chinese Medicine, Shanghai 200071, China; ^2^Shanghai Public Health Clinical Center, Fudan University, Shanghai 201508, China; ^3^Department of Traditional Chinese Medicine, Medical College of Xiamen University, Xiamen 361005, China

## Abstract

**Background and Aims:**

Traditional Chinese medicine (TCM) therapy for hepatocellular carcinoma remains controversial. This study aimed to evaluate the efficacy and safety of TCM regimens in HCC treatment.

**Methods:**

Randomized controlled trials (RCTs) up to June 1, 2016, of the TCM treatment for hepatocellular carcinoma were systematically identified in PubMed, CNKI, Ovid, Embase, Web of Science, Wanfang, VIP, CBM, AMED, and Cochrane Library databases.

**Results:**

A total of 1010 and 931 patients in 20 RCTs were randomly treated with add-on TCM therapy and conventional therapy, respectively. The additional use of TCM significantly improved six-month, one-year, two-year, and three-year overall survival rates in HCC cases (RR = 1.3, *P* = 0.01; RR = 1.38, *P* = 0.0008; RR = 1.44, *P* < 0.0001; RR = 1.31, *P* = 0.02, resp.). Add-on TCM therapy significantly increased PR rate and total response rate (tRR) and reduced PD rate compared to those in control group (34.4% versus 26.3%, RR = 1.30, *P* = 0.002; 41.6% versus 31.0%, RR = 1.30, *P* < 0.0001; and 16.6% versus 26.5%, RR = 0.64, *P* < 0.0001, resp.). Additionally, TCM combination therapy significantly increased the quality of life (QOL) improvement rate and reduced adverse events including leukopenia, thrombocytopenia, anemia or erythropenia, liver injury, and gastrointestinal discomfort in HCC patients (all *P* < 0.05).

**Conclusion:**

Add-on therapy with TCM could improve overall survival, increase clinical tumor responses, lead to better QOL, and reduce adverse events in hepatocellular carcinoma.

## 1. Introduction

Primary liver cancer is the sixth most common cancer and the third most common cause of cancer-related deaths. 70%~90% primary liver cancers occurring worldwide are hepatocellular carcinoma (HCC), which is the fastest growing cause of cancer-related death globally [1, 2]. Recent epidemiology data revealed that liver cancer might account for more cancer-related deaths worldwide [3]. HCC has a 5-year survival rate of only 14% approximately [4]. Most HCCs are diagnosed at an intermediate to advanced stage, at which point surgical treatment and/or chemical embolism are no longer feasible [5]. Therefore, to improve outcome of HCC patients, an alternative or novel approach is required.

Previous report showed a large prevalence of a diversity of traditional Chinese medicine (TCM) clinical application for cancer patients [6]. Sufficient evidence has demonstrated that natural compounds with various types of medicinal ingredients can substantially inhibit tumor formation [7]. Many clinical articles have reported that TCM or TCM plus chemotherapy can significantly alleviate symptoms, stabilize tumor size, reinforce the constitution, enhance therapy tolerance and immunological function, obviously reduce the incidence rate of adverse events, and prolong patients' survival duration for unresectable HCC [8–11].

Unfortunately, reporting of RCTs on treatment of HCC with TCM is still in low quality, not meeting the CONSORT and TREND statement. High quality of evidence based on the existing clinical information is still unavailable [6, 12]. A recent meta-analysis also announced that many RCTs of TCM therapy in HCC are not, in fact, randomized [13]. Thus, only RCTs reported randomized methods were included in our current meta-analysis. The purpose of this study is to systematically review and meta-analyze data from RCTs for evidence on the efficacy and safety of add-on therapy with TCM in the treatment of HCC.

## 2. Materials and Methods

### 2.1. Search Strategy and Study Selection

We searched PubMed, Chinese National Knowledge Infrastructure (CNKI) Database, Wanfang Database, Chinese Biomedical (CBM) Database, Chinese Science and Technology Periodical Database (VIP), Allied and Complementary Medicine Database (AMED), Ovid, Embase, Web of Science, and Cochrane Library databases until June 1, 2016. The following medical subject headings were used: “hepatocellular carcinoma;” “primary liver cancer;” “Traditional Chinese Medicine;” “alternative medicine;” “complementary medicine;” “Chinese herbal medicine;” “herb/herbal;” and “decotion/formulation.” Electronic searches were supplemented with manual searches of reference lists used in all of the retrieved review articles, primary studies, and abstracts from meetings to identify other studies not found in the electronic searches. Literature was searched by two authors (Z Yang and X Liao) independently.

Two authors independently selected trials and discussed with each other when inconsistencies were found. Articles that satisfy the following criteria were included: (1) for study types, RCTs with randomized method; (2) for participants, HCCs; (3) for interventions, TCMs compared with placebo or no treatment; in addition, any cointervention had to be the same in both groups except for the TCM formulation; (4) for outcome, overall survival and/or solid tumors responses; and (5) available full texts. If the duration and sources of study population recruitment overlapped by more than 30% in two or more papers by the same authors, we only included the most recent study or the study with the larger number of HCC patients. Studies were excluded if they meet the following criteria: (1) studies “so-called” randomized without randomized methods; (2) studies without control subjects or control participants receiving TCM treatment including herbal medicine and acupuncture; (3) studies reporting only laboratory values and/or symptom improvement rather than survival outcomes and clinical responses.

### 2.2. Data Extraction and Methodological Quality Assessment

Two researchers independently read the full texts and extracted the following contents: publication data; study design; sample size; patient characteristics; treatment protocol; and outcome measures. The methodological qualities of the included RCTs were assessed according to Cochrane Collaboration's Tool described in Handbook version 5.1.0 [14]. Two authors (Z Yang and X Liao) independently assessed quality, and inconsistency was discussed with other reviewer-authors (Y Yu and X Chen) who acted as arbiters.

### 2.3. Definitions

All the diagnosis should be according to guidelines. The primary outcome overall survival was defined as the time from HCC diagnosis until the death due to any cause. Solid tumor response is categorized as complete response (CR), partial response (PR), stable disease (SD), progressive disease (PD), and CR + PR as a proportion for total response rate (tRR) according to the World Health Organization (WHO) criteria [15] or the Response Evaluation Criteria In Solid Tumors (RECIST) guidelines [16, 17]. Karnofsky performance status (KPS) [18] and adverse events were also measured in our study.

### 2.4. Statistical Methods

The effect measures of interest were risk ratios (RRs) and the corresponding 95% confidence intervals (CIs). Heterogeneity across studies was informally assessed by visually inspecting forest plots and formally estimated by Cochran's *Q* test in which chi-square distribution is used to make inferences regarding the null hypothesis of homogeneity (considered significant at *P* < 0.10). A rough guide to our interpretation of *I*^2^ was listed as follows:

- 0% to 40% shows that heterogeneity may not be important.
- 30% to 60% corresponds to moderate heterogeneity.
- 50% to 90% exhibits substantial heterogeneity.
- 75% to 100% indicates considerable heterogeneity [14, 19].

If the eligibility of some studies in the meta-analysis was uncertain because of missing information, a sensitivity analysis was performed by conducting the meta-analysis twice: in the first meta-analysis, all of the studies were included; in the second meta-analysis, only those that were definitely eligible were included. A fixed-effects model was used initially for our meta-analyses; a random-effects model was then used in the presence of heterogeneity. Description analysis was performed when quantitative data could not be pooled. Review Manager version 5.1 software was used for data analysis.

## 3. Results

### 3.1. Study and Patient Characteristics

**Figure 1:**
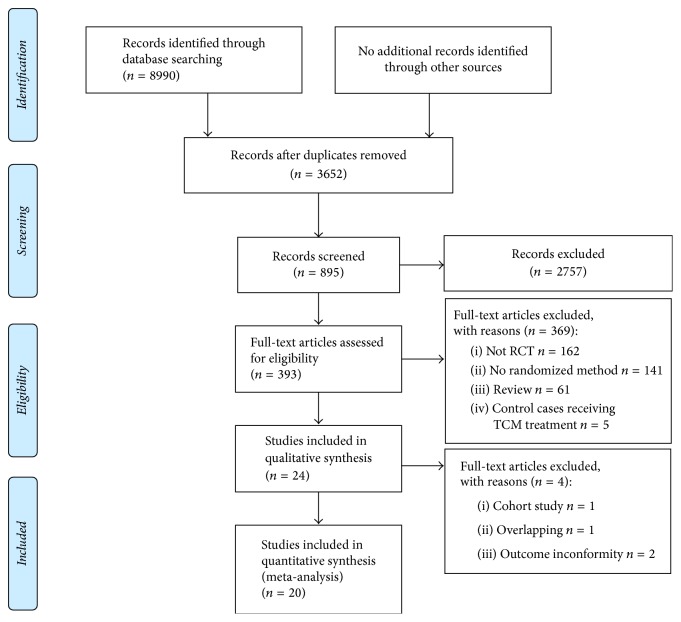
Flow diagram of study selection.

Totally, 8990 abstracts were reviewed; among these articles, 393 were retrieved that are closely related to the current subject. The study selection process was summarized in Figure 1. Finally, 20 RCTs [20–39] were included in this meta-analysis. The baseline characteristics of included studies are described in Table 1.

### 3.2. Methodological Quality Assessment

**Figure 2:**
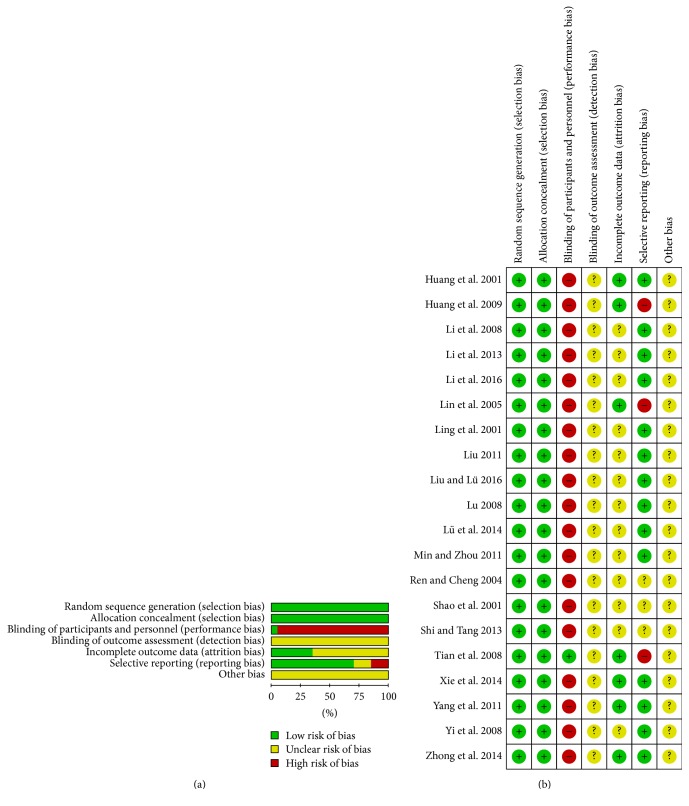
Risk of bias graph (a) and risk of bias summary (b).

The methods of randomization were described adequately in all studies [20–39], which were considered as random number table [20, 21, 23, 24, 27, 28, 30–32, 34], sealed envelopes [22, 26, 37–39], randomized block [25, 35], draw method [29, 36], and randomization according to hospitalized date [33]. We hence considered low risks in terms of selection bias. Except for study reported by Tian et al. [35], blind-methods of other studies were not available, which were considered high risk in terms of performance bias. Detection bias was unclear in all studies with no presenting of blinding of outcome assessment. Less than 15% of participants were lost to follow-up in the three studies [20, 21, 25, 35–37, 39]; these parameters were considered low risk in terms of incomplete outcome data. Selective reporting was found in three studies [21, 25, 35] because these researches failed to present the clinical data of participants in ITT analysis. Other potential biases were unclear in these trials (Figure 2).

### 3.3. Overall Survival

**Figure 3:**
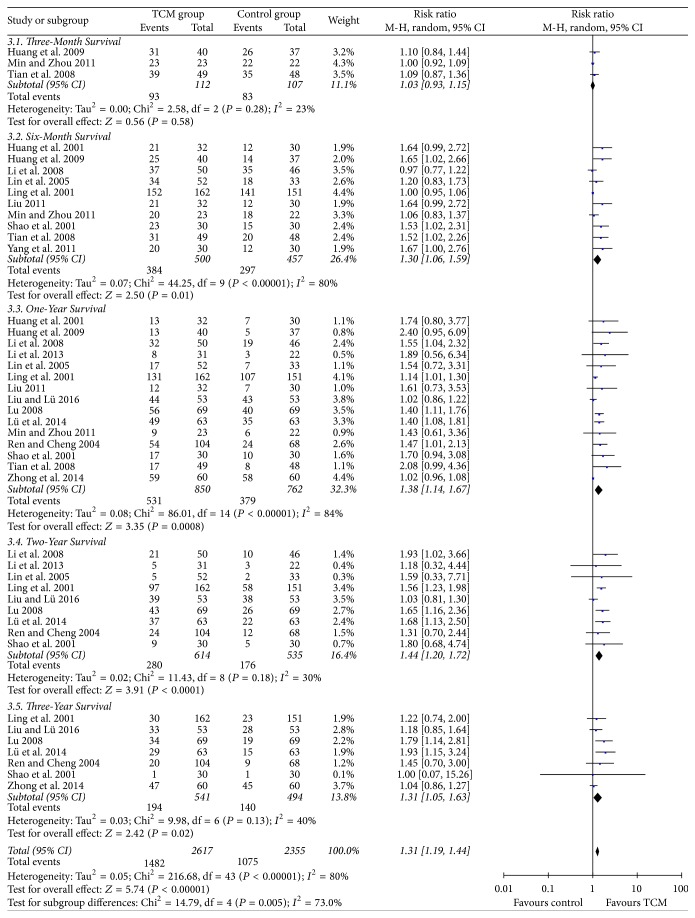
Overall surviving comparison.

No heterogeneity was found among the included studies [21, 31, 35], which reported three-month survival in the two groups. No significance of three-month survival was found in HCC patients between TCM group and control group (RR = 1.03, 95% CI = 0.93–1.15, *P* = 0.58, Figure 3(3.1)). Heterogeneity was significant when we compared six-month survival and one-year survival (*P* < 0.00001, *I*^2^ = 80% and *P* < 0.00001, *I*^2^ = 84%, resp.). As shown in Figure 3, TCM therapy could significantly prolong six-month survival and one-year survival of HCC patients compared to control (RR = 1.30, 95% CI = 1.06–1.59, and *P* = 0.01 and RR = 1.38, 95% CI = 1.14–1.67, and *P* = 0.0008, resp., Figure 3(3.2 and 3.3)).

No heterogeneity was found between studies comparing two-year survival and three-year survival between the two groups (*P* = 0.18, *I*^2^ = 30% and *P* = 0.13, *I*^2^ = 40%, resp.). Meta-analysis of RCTs [22, 23, 25, 26, 28–30, 32, 33] using a random-effects model showed that the two-year survival rate of HCC patients in TCM group was significantly higher than that in control group [280/614 (45.6%) versus 176/535 (32.9%), RR = 1.44, 95% CI = 1.20–1.72, and *P* < 0.0001, Figure 3(3.4)]. Similarly, the three-year survival rate of HCC patients receiving TCM therapy was significantly higher than that in control group [194/541 (35.9%) versus 140/494 (28.3%), RR = 1.31, 95% CI = 1.05–1.63, and *P* = 0.02, Figure 3(3.5)].

### 3.4. CR, PR, SD, PD, and tRR

As shown in Table 2, no heterogeneity was among comparisons of CR, PR, SD, PD, and tRR. Thus, a fixed-effects model was used. The CR rate of HCC patients in TCM group was higher than that in control group, but no statistical difference was found (RR = 1.47, 95% CI = 0.96–2.24, and *P* = 0.07, Figure S1; see Supplementary Material available online at https://doi.org/10.1155/2017/3428253). However, meta-analysis of RCTs [20, 22–25, 27, 29, 30, 32, 34–36, 38] demonstrated that HCC patients receiving TCM therapy achieved significantly higher PR rate and tRR than those in control group (34.4% versus 26.3%, RR = 1.30, 95% CI = 1.10–1.53, and *P* = 0.002 and 41.6% versus 31.0%, RR = 1.30, 95% CI = 1.16–1.53, and *P* < 0.0001, resp., Figure S2 and Figure S5). In contrast, HCC patients in TCM group suffered from lower PD rate significantly than those in control group (16.6% versus 26.5%, RR = 0.64, 95% CI = 0.52–0.80, and *P* < 0.0001, Figure S4). No statistical significance was found when we compared SD rate of HCC patients between TCM group and control group (42.6% versus 43.7%, RR = 0.95, 95% CI = 0.84–1.08, and *P* = 0.47, Figure S3).

### 3.5. Quality of Life (QOL)

**Figure 4:**
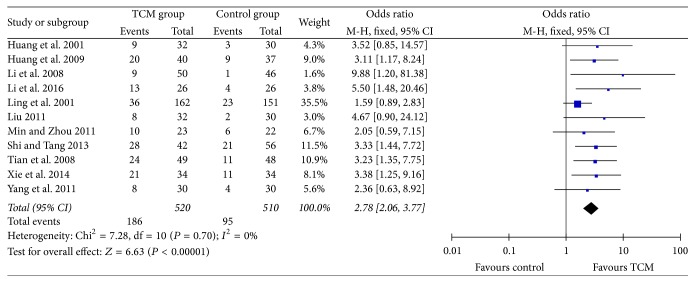
Improvement rate of quality of life according to KPS scores.

In this meta-analysis, KPS scores increasing more than 10 after treatment compared to that before treatment was considered improvement in QOL. 11 RCTs [20–22, 24, 26, 27, 31, 34–37] reported QOL assessment according to KPS scores, with no significance of heterogeneity which existed (*P* = 0.70, *I*^2^ = 0%). As shown in Figure 4, the QOL improvement rate of HCC patients in TCM group was significantly higher than that in control group [186/520 (35.8) versus 95/510 (18.6), RR = 2.78, 95% CI = 2.06–3.77, and *P* < 0.00001].

### 3.6. Adverse Events

**Figure 5:**
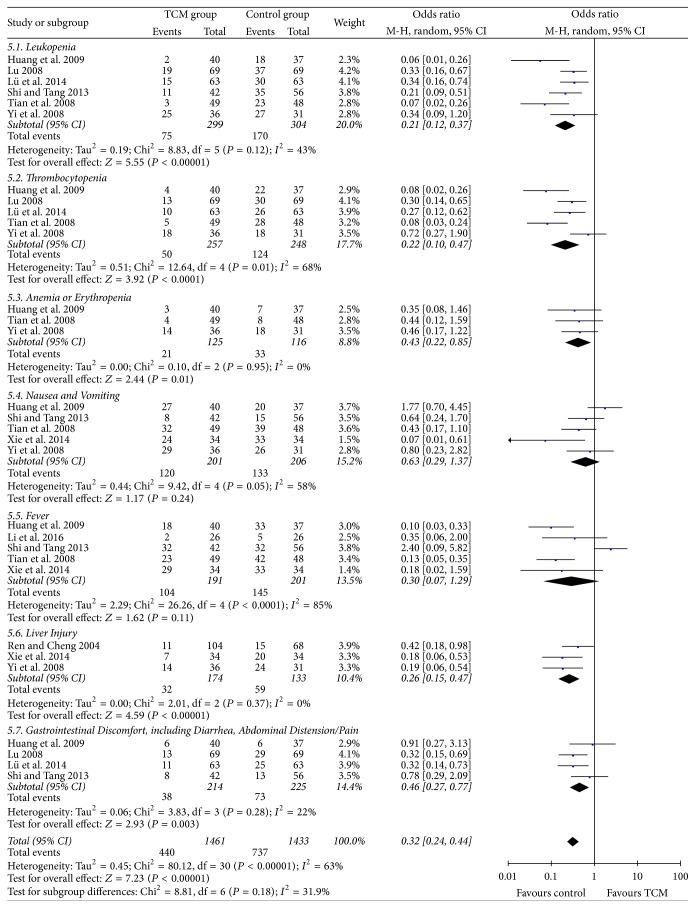
Adverse events incidence.

Nine RCTs [21, 24, 29, 30, 32, 34–36, 38] reported the adverse events incidence of HCC patients. The most frequent adverse events were leukopenia, thrombocytopenia, anemia/erythropenia, nausea, vomiting, fever, liver injury, and gastrointestinal discomfort. Meta-analysis indicated that HCC patients in control group had significantly higher risk of suffering from leukopenia, thrombocytopenia, anemia/erythropenia, liver injury, and gastrointestinal discomfort than those receiving TCM therapy (55.9% versus 25.1%, 50.0% versus 19.5%, 28.4% versus 16.8%, 44.4% versus 18.4%, and 32.4% versus 17.8%, respectively, all *P* < 0.05, Figure 5). No statistical significance of nausea/vomiting and fever was found between the two groups (*P* = 0.24 and *P* = 0.11, resp., Figure 5).

## 4. Discussion

Most newly diagnosed HCC cases are at an intermediate advanced stage, and the therapeutic options are limited to palliative approaches using TACE or chemotherapeutic agents [5, 40]. Even worse, many patients poorly respond to TACE or suffer from poor outcomes and side effects with conventional systemic cytotoxic chemotherapy [40], leading to disappointing results of systemic chemotherapies and a poor prognosis. Therefore, novel therapeutic strategies are essential to improve the clinical management of patients with HCC.

With a long history of clinical use, essential components of TCM have gradually become a common used treatment for cancer in China [41]. In particular, TCM has been used to treat HCC extensively and it can be used throughout the whole course of HCC [42]. In the past decades, many compounds derived from Chinese herbals of both preclinical and clinical researches have shown promising potentials in novel anti-HCC natural product development [43]. Previous studies indicated that the effect of TCM has targeted the stimulation of the host immune response for cytotoxic activity against liver cancer by inhibiting proliferation and promoting apoptosis of tumor cells [7, 44], thereby improving survival and alleviating palliative approaches-related side effects in HCC patients [45–47].

This meta-analysis summarized evidence on the effects of TCM therapy for HCC patients, on top of conventional treatment. For survival, it is observed that the additional use of TCM significantly improved six-month, one-year, two-year, and three-year survival rates in HCC cases. Additionally, TCM combination therapy could increase PR rate and tRR and reduce PD rate in this population. Given above, results from our study demonstrated add-on benefits of TCM in improving outcomes of HCC patients. As the molecular pathogenesis of HCC is highly associated with multigene, multifactor, and multistep processes and is quite complicated, add-on TCM therapy combined with other therapeutic options has a promising potential for its multilevel, multitarget, and coordinated intervention effects against HCC [43]. Many active compounds from TCM have shown their noticeable potentials in inhibiting the promotion, proliferation, angiogenesis, and metastasis of HCC [43, 44], which may contribute to good tumor response and survival in clinical practice. Although the mechanisms of TCM components in anti-HCC were well reviewed before [43], further in-depth mechanistic studies and well-designed clinical trials are warranted.

Previous work has suggested that QOL is an important predictor of survival for cancer patients [48]. Although more sophisticated approaches of QOL measurement were developed, the KPS scores are still widely recognized as a tool for the assessment of the functional status of cancer patients and highly reliable [49]. Based on the evidence we identified, TCM combination therapy may be considered as an alternative option to improve QOL in HCC patients. Previously, KPS as a predictor of survival has been demonstrated in patients with different kind of cancers [48, 49], and few studies focused on the relationship between KPS scores and HCC survival. Whether KPS has a role in predicting HCC outcomes should be focused on in future studies.

Evidence of this meta-analysis also showed that the combination of TCM and chemotherapy significantly reduced adverse events including leukopenia, thrombocytopenia, anemia or erythropenia, liver injury, and gastrointestinal discomfort in HCC patients. However, because of the toxic effects of chemotherapy and anticancer drugs on normal cells and tissues, anticancer drugs and approaches cause many side effects and adverse events with various symptoms, including hematocytopenia, gastrointestinal discomfort (nausea, vomiting, anorexia, and diarrhea), and liver injury. These side effects often influence patients' QOL and sometimes make the chemotherapy discontinued [50, 51]. Consistent with our results, growing evidences suggest that TCM appears to have beneficial effects for prevention and improvement of several chemotherapy-induced side effects [52, 53], leading to better outcomes in this population.

This meta-analysis had the following limitations. First, majority of the included studies had small samples, with mid- to low-quality designs. Second, all included studies were conducted in China. According to our experience, only positive results are published in Chinese medical journals. We cautiously drew the conclusion that publication bias might have been present in this meta-analysis. Third, most included studies failed to address blinding assessment, which may influence the objectivity of HCC outcomes. High-quality, well-designed, large sample trials focused on the efficacy and safety of TCM therapy for HCC should be performed in the future.

In conclusion, add-on therapy with TCM could improve overall survival, increase clinical tumor responses, and reduce adverse events in hepatocellular carcinoma. Previous surveys indicated that the trend of TCM use in patients with cancer is on the rise. Surveys have also found that many cancer patients were more inclined to use TCM therapies in combination with conventional therapy rather than in lieu of conventional therapy [54]. Thus, investigating the combined use of TCM and conventional therapy in the oncology setting is urgently essential for practitioners. Evidence-based approaches in the clinic have to be supplemented by experimental studies to unravel cellular and molecular modes of action of TCM treatments [45].

## Supplementary Material

Figure S1: Complete response (CR) rate comparison between TCM group and control group.
Figure S2: Partial response (PR) rate comparison between TCM group and control group.
Figure S3: Stable disease (SD) rate comparison between TCM group and control group.
Figure S4: Progressive disease (PD) rate comparison between TCM group and control group.
Figure S5: Total response rate (tRR) comparison between TCM group and control group.

## Figures and Tables

**Table 1:** Characteristics of studies included in the meta-analysis.

Study	Number of cases		Control regimen	Chemo times	TCM intervention	TCM duration (days)	HCC staging	Child-Pugh score	KPS score	Randomized method
	Treatment	Control								
Huang et al. 2001 [20]	32	30	Palliatively supporting therapy	None	Jianpi Xiaoji oral liquid	30	III, IV	A, B, C	≥60	Random number table
Huang et al. 2009 [21]	40	37	TACE	1~2	Ganji decoction	28~42	II, III	A, B	≥60	Random number table
Li et al. 2008 [22]	50	46	TACE	2~7	Chinese toad bufotoxin injection	28~112	*Okuda*I, II, III	A, B	≥60	Sealed envelopes
Li et al. 2013 [23]	31	22	TACE	3	Hydroxycamptothecin and Chinese herbal compound	84	II, III	NA	NA	Random number table
Li et al. 2016 [24]	26	26	TACE	NA	Aidi injection	30	III, IV	NA	30~60	Random number table
Lin et al. 2005 [25]	52	33	TACE	2	Hydroxycamptothecin and Shentao Ruangan pill	56	II, III	A, B	≥60	Randomized block
Ling et al. 2001 [26]	162	151	TACE/PEI	NA	Sisheng decoction/Chinese toad bufotoxin injection/norcantharidin tablets	Irregular	II, III	NA	NA	Sealed envelopes
Liu 2011 [27]	32	30	Palliatively supporting therapy	None	Chinese herbal compound	21	III, IV	A, B, C	≥60	Random number table
Liu and Lü 2016 [28]	53	53	TACE + PMCT	3	Chinese herbal compound	90~135	NA	A, B	NA	Random number table
Lu 2008 [29]	69	69	TACE	NA	Aidi injection	20	NA	NA	60~90	Draw method
Lü et al. 2014 [30]	63	63	TACE	NA	Shenyi capsules	60	NA	NA	60~90	Random number table
Min and Zhou 2011 [31]	23	22	Palliatively supporting therapy	None	Chinese herbal compound	84	II, III	A, B	72 ± 8	Random number table
Ren and Cheng 2004 [32]	104	68	TACE	NA	Chinese herbal compound	≥90	II, III	NA	NA	Random number table
Shao et al. 2001 [33]	30	30	TACE	2~10	Chinese herbal compound	180~300	II, III	NA	NA	According to hospitalized date
Shi and Tang 2013 [34]	42	56	TACE	NA	Kang'ai injection and Carapacis Trionycis Bolus	56	NA	A, B	≥60	Random number table
Tian et al. 2008 [35]	49	48	TACE	NA	Chinese herbal compound	28	II, III	A, B	≥60	Randomized block, single blind
Xie et al. 2014 [36]	34	34	TACE	NA	Chinese herbal compound	40	NA	A, B	≥60	Draw method
Yang et al. 2011 [37]	30	30	TACE	NA	Aidi injection	30	NA	A, B, C	≥70	Sealed envelopes
Yi et al. 2008 [38]	28	23	TACE	3	Kang'ai injection	45	II, III	NA	≥60	Sealed envelopes
Zhong et al. 2014 [39]	60	60	Hepatectomy	None	Chinese herbal compound	365	I~IIIa	A, B	NA	Sealed envelopes

TACE, transcatheter arterial chemoembolization; PEI, percutaneous ethanol injection; PMCT, percutaneous microwave coagulation therapy; KPS, Karnofsky performance status.

**Table 2:** Solid tumor responses comparisons of HCC patients.

Comparisons	Studies	Groups	Clinical responses (%)	Heterogeneity				RR	95% CI	*P* value
				Chi^2^	df	*P*	*I* ^2^ (%)			
Complete response (CR)	[22, 23, 29, 30, 32, 34–36]	Treatment	44/442 (10.0)	3.07	7	0.88	0	1.47	0.96–2.24	0.07
		Control	26/406 (6.4)							
Partial response (PR)	[20, 22–25, 27, 29, 30, 32, 34–36, 38]	Treatment	210/610 (34.4)	9.96	12	0.62	0	1.30	1.10–1.53	0.002
		Control	145/551 (26.3)							
Stable disease (SD)	[20, 22–25, 27, 29, 30, 32, 34–36, 38]	Treatment	260/610 (42.6)	15.89	12	0.2	24	0.95	0.84–1.08	0.47
		Control	241/551 (43.7)							
Progressive disease (PD)	[20, 22–25, 27, 29, 30, 32, 34–36, 38]	Treatment	101/610 (16.6)	9.22	12	0.68	0	0.64	0.52–0.80	<0.0001
		Control	146/551 (26.5)							
Total response rate (tRR)	[20, 22–25, 27, 29, 30, 32, 34–36, 38]	Treatment	254/610 (41.6)	13.56	12	0.33	12	1.3	1.16–1.53	<0.0001
		Control	171/551 (31.0)							
