# Supplementary figures and images for: Add-On Therapy with Traditional Chinese Medicine Improves Outcomes and Reduces Adverse Events in Hepatocellular Carcinoma: A Meta-Analysis of Randomized Controlled Trials
Source: Evid Based Complement Alternat Med. 2017 Jun 7;2017:3428253. doi: 10.1155/2017/3428253 (PMC5478821; doi:10.1155/2017/3428253)

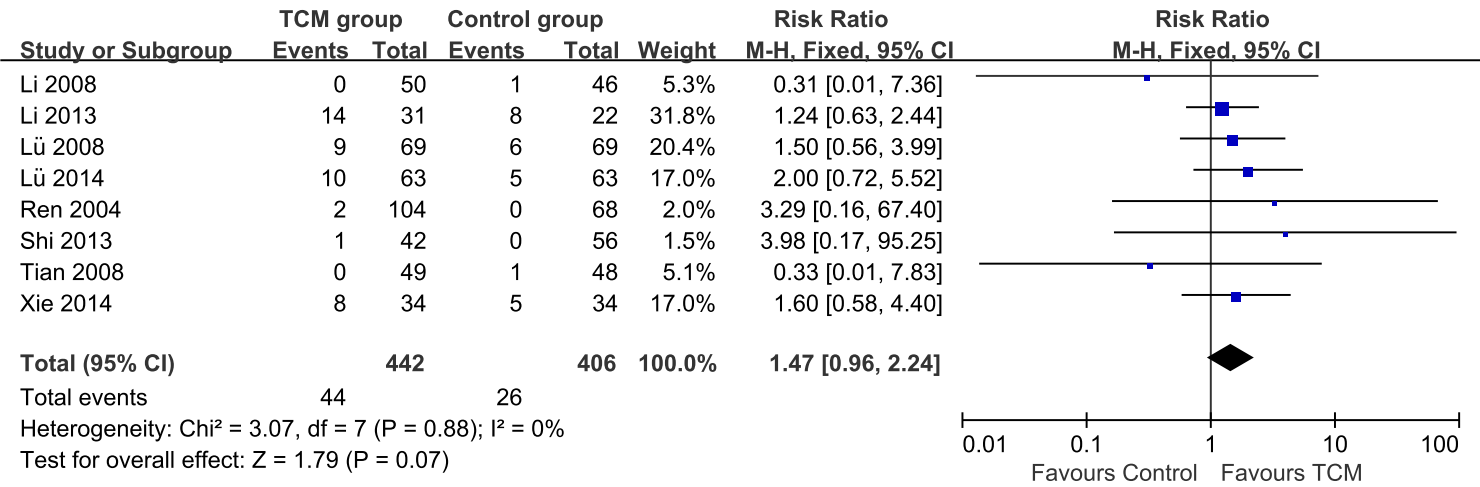

Supplement: Supplementary file 1 — Figure S1: Complete response (CR) rate comparison between TCM group and control group. Figure S2: Partial response (PR) rate comparison between TCM group and control group. Figure S3: Stable disease (SD) rate comparison between TCM group and control group. Figure S4: Progressive disease (PD) rate comparison between TCM group and control group. Figure S5: Total response rate (tRR) comparison between TCM group and control group. [file 3428253.f1.zip › Figure S1_Complete Response_ECAM_1898824.pdf]

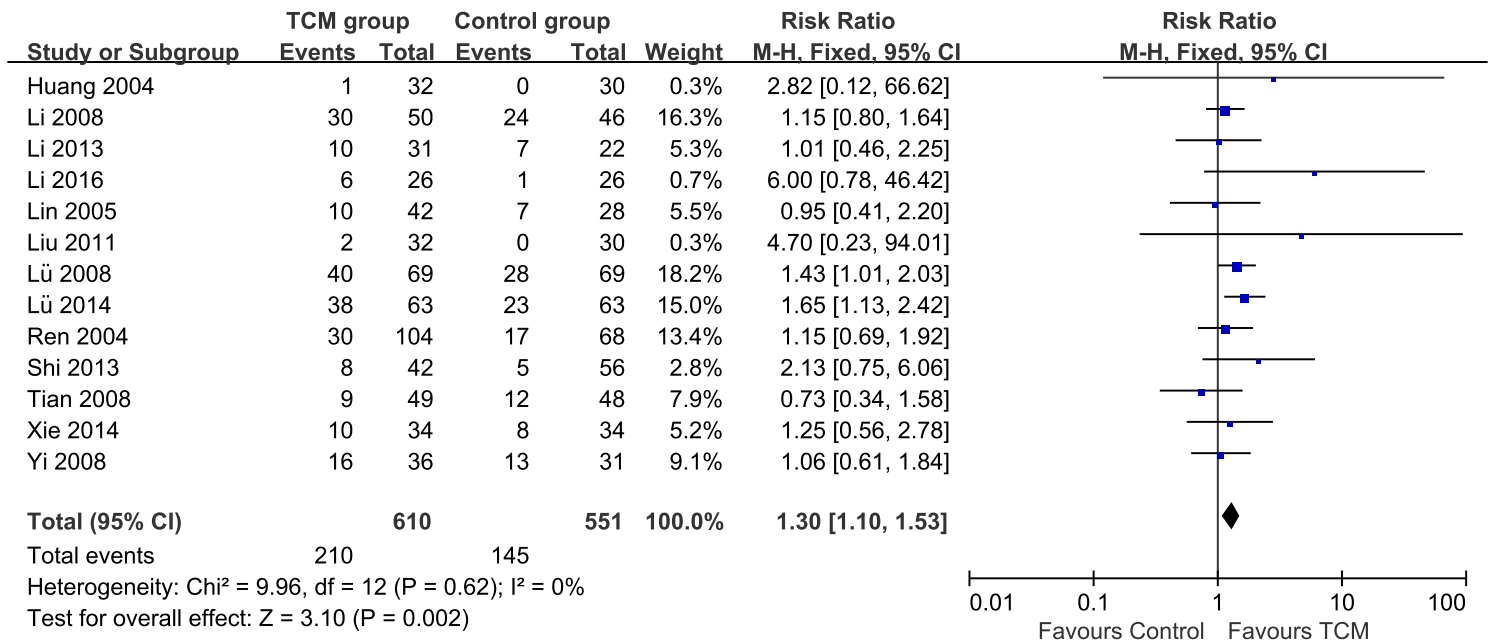

Supplement: Supplementary file 1 — Figure S1: Complete response (CR) rate comparison between TCM group and control group. Figure S2: Partial response (PR) rate comparison between TCM group and control group. Figure S3: Stable disease (SD) rate comparison between TCM group and control group. Figure S4: Progressive disease (PD) rate comparison between TCM group and control group. Figure S5: Total response rate (tRR) comparison between TCM group and control group. [file 3428253.f1.zip › Figure S2_Partial response_ECAM_1898825.pdf]

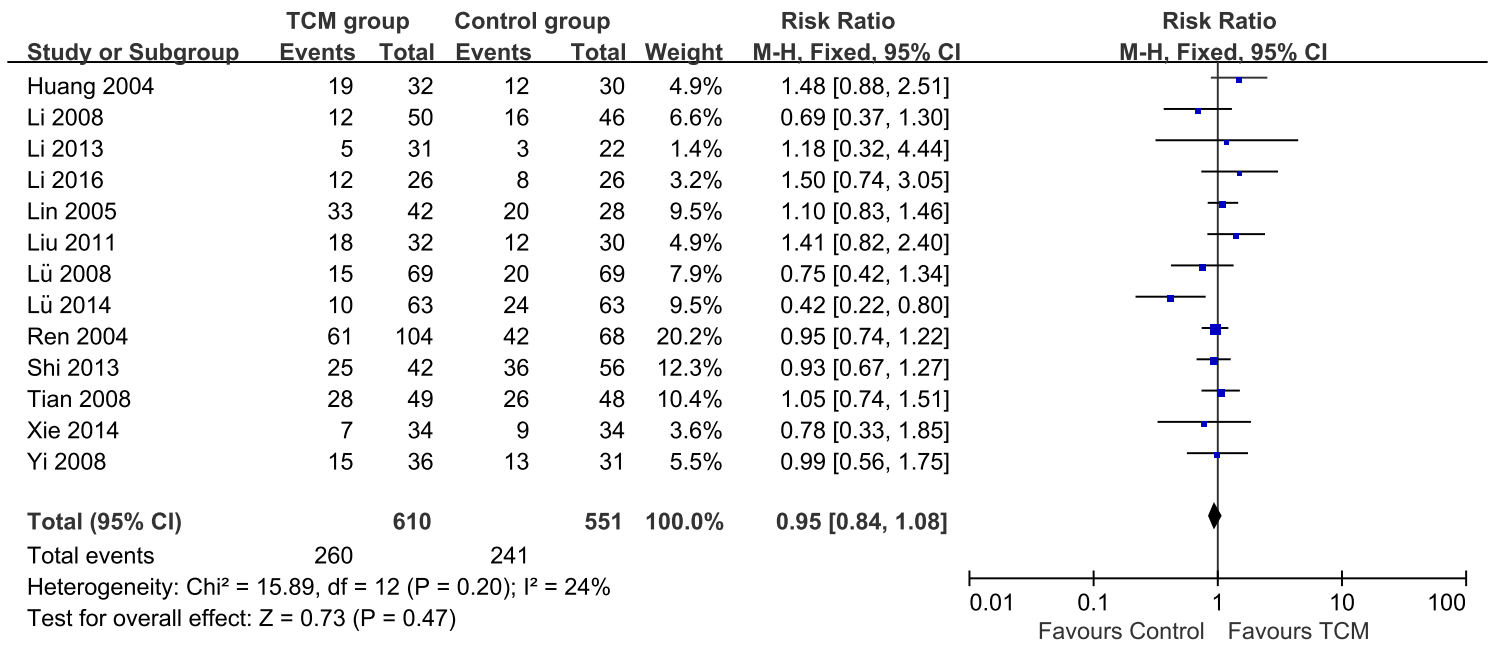

Supplement: Supplementary file 1 — Figure S1: Complete response (CR) rate comparison between TCM group and control group. Figure S2: Partial response (PR) rate comparison between TCM group and control group. Figure S3: Stable disease (SD) rate comparison between TCM group and control group. Figure S4: Progressive disease (PD) rate comparison between TCM group and control group. Figure S5: Total response rate (tRR) comparison between TCM group and control group. [file 3428253.f1.zip › Figure S3_Stable disease_ECAM_1898827.pdf]

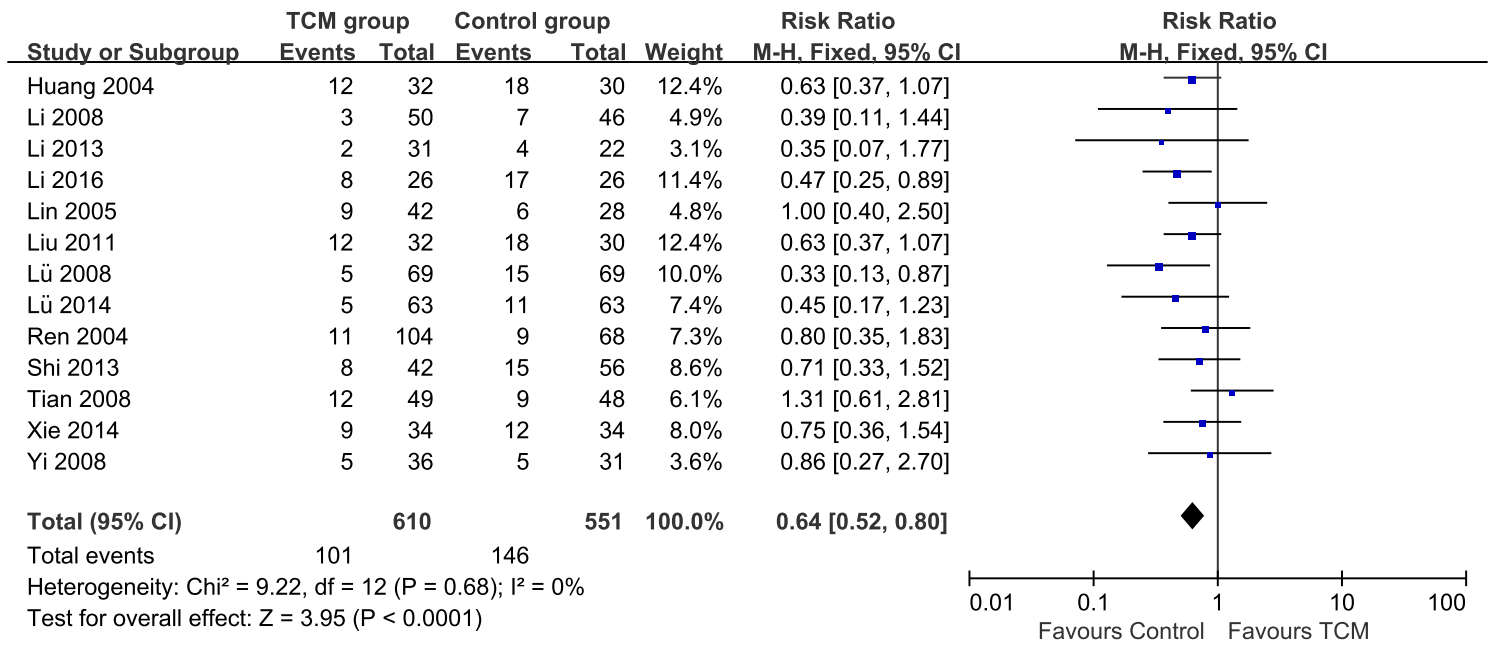

Supplement: Supplementary file 1 — Figure S1: Complete response (CR) rate comparison between TCM group and control group. Figure S2: Partial response (PR) rate comparison between TCM group and control group. Figure S3: Stable disease (SD) rate comparison between TCM group and control group. Figure S4: Progressive disease (PD) rate comparison between TCM group and control group. Figure S5: Total response rate (tRR) comparison between TCM group and control group. [file 3428253.f1.zip › Figure S4_Progressive disease_ECAM_1898828.pdf]

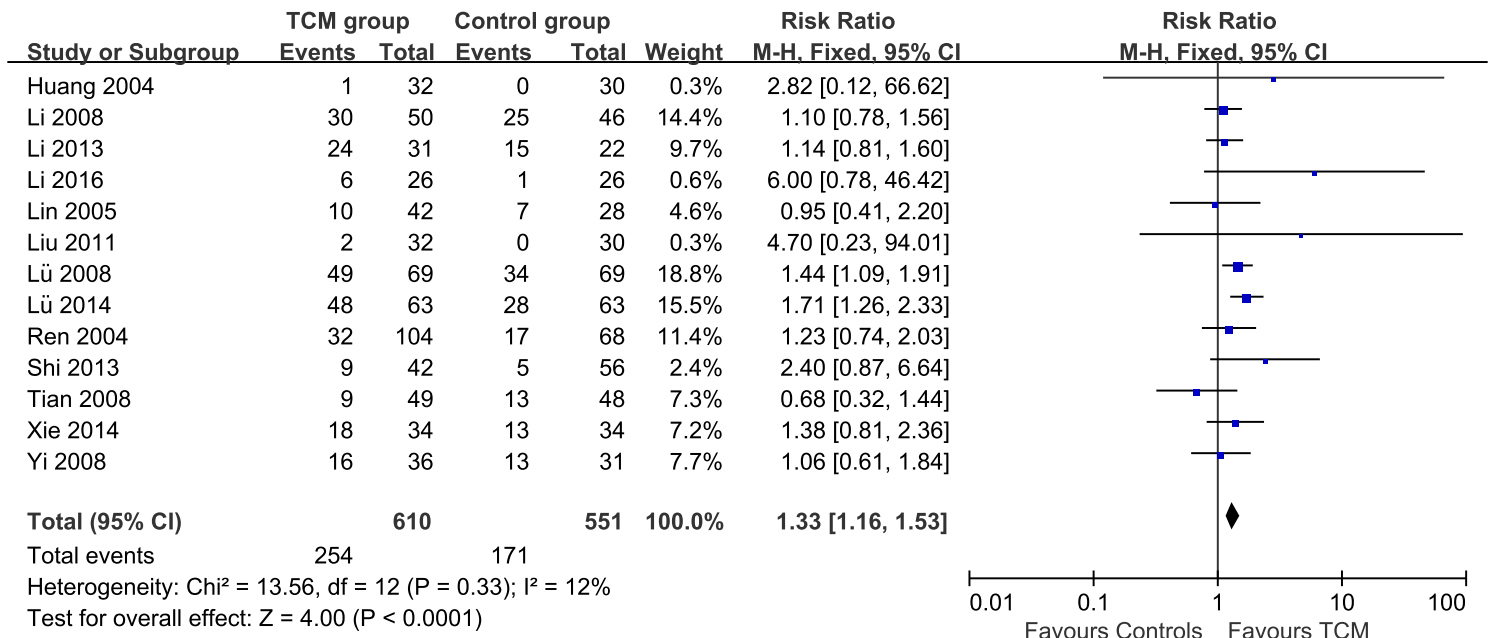

Supplement: Supplementary file 1 — Figure S1: Complete response (CR) rate comparison between TCM group and control group. Figure S2: Partial response (PR) rate comparison between TCM group and control group. Figure S3: Stable disease (SD) rate comparison between TCM group and control group. Figure S4: Progressive disease (PD) rate comparison between TCM group and control group. Figure S5: Total response rate (tRR) comparison between TCM group and control group. [file 3428253.f1.zip › Figure S5_Response Rate_ECAM_1898829.pdf]
